# RETRACTION: Arctigenin, a Potent Ingredient of *Arctium lappa* L., Induces Endothelial Nitric Oxide Synthase and Attenuates Subarachnoid Hemorrhage‐Induced Vasospasm through PI3K/Akt Pathway in a Rat Model

**DOI:** 10.1155/bmri/9836896

**Published:** 2026-04-03

**Authors:** BioMed Research International

RETRACTION: C.‐Z. Chang, S.‐C. Wu, C.‐M. Chang, C.‐L. Lin, and A.‐L. Kwan, “Arctigenin, a Potent Ingredient of *Arctium lappa* L., Induces Endothelial Nitric Oxide Synthase and Attenuates Subarachnoid Hemorrhage‐Induced Vasospasm through PI3K/Akt Pathway in a Rat Model,” *BioMed Research International* 2015, 490209; https://doi.org/10.1155/2015/490209.

The above article, published online on 11 October 2015 in Wiley Online Library (wileyonlinelibrary.com), has been retracted by agreement between the journal’s Editor‐in‐Chief, Yoel A. Klug; and John Wiley & Sons Ltd. A third party reported that images in Figure 1 in this article had been published in several other articles by some of the same authors. Each image was reported differently in the various publications.

The authors did not respond to an inquiry and request for original data by the publisher.

The retraction has been agreed to because the evidence of image duplication fundamentally compromises the editors’ confidence in the results presented. The authors have been informed of the retraction.
